# Decreasing trends in cholangiocarcinoma incidence and relative survival in Khon Kaen, Thailand: An updated, inclusive, population-based cancer registry analysis for 1989–2018

**DOI:** 10.1371/journal.pone.0246490

**Published:** 2021-02-16

**Authors:** Supot Kamsa-ard, Chalongpon Santong, Siriporn Kamsa-ard, Vor Luvira, Varisara Luvira, Krittika Suwanrungruang, Vajarabhongsa Bhudhisawasdi

**Affiliations:** 1 Department of Epidemiology and Biostatistics, Faculty of Public Health, Khon Kaen University, Khon Kaen, Thailand; 2 ASEAN Cancer Epidemiology and Prevention Research Group, Khon Kaen University, Khon Kaen, Thailand; 3 Cancer Unit, Srinagarind Hospital, Faculty of Medicine, Khon Kaen University, Khon Kaen, Thailand; 4 Department of Surgery, Faculty of Medicine, Khon Kaen University, Khon Kaen, Thailand; 5 Department of Community Medicine, Faculty of Medicine, Khon Kaen University, Khon Kaen, Thailand; Dokkyo Medical University, JAPAN

## Abstract

**Background:**

Cholangiocarcinoma (CCA) is a leading cause of cancer death in northeastern Thailand. We reported on the incidence of CCA using only one method. In the current study, we used three different statistical methods to forecast future trends and estimate relative survival.

**Methods:**

We reviewed the CCA cases diagnosed between 1989 and 2018 recorded in the population-based Khon Kaen Cancer Registry (KKCR). Annual percent change (APC) was calculated to quantify the incidence rate trends using Joinpoint regression. Age-period-cohort models (APC model) were used to examine the temporal trends of CCA by age, calendar year, and birth cohort. We projected the incidence of CCA up to 2028 using three independent approaches: the Joinpoint, Age-period-cohort, and Nordpred models. Survival assessments were based on relative survival (RS).

**Results:**

The respective APC in males and females decreased significantly (-3.1%; 95%CI: -4.0 to -2.1 and -2.4%; 95%CI: -3.6 to -1.2). The APC model—AC-P for male CCA—decreased according to a birth-cohort. The CCA incidence for males born in 1998 was 0.09 times higher than for those born in 1966 (Incidence rate ratios, IRR = 0.09; 95%CI: 0.07 to 0.12). The relative incidence for female CCA similarly decreased according to a birth-cohort (IRR = 0.11; 95%CI: 0.07 to 0.17). The respective projection for the age-standardized rate for males and females for 2028 will be 7.6 per 100,000 (102 patients) and 3.6 per 100,000 (140 patients). The five-year RS for CCA was 10.9% (95%CI: 10.3 to 11.6).

**Conclusion:**

The incidence rate of CCA has decreased. The projection for 2028 is that the incidence will continue to decline. Nevertheless, the survival of patients with CCA remains poor.

## Introduction

Globally, liver cancer is one of the most common cancers. In 2020, there were an estimated 905,677 (4.7%) new liver cancer cases and 830,480 (8.3%) deaths worldwide. Sixty percent of cases lived in a developing country [1, 2]. Liver cancer rates are the highest in East and Southeast Asia and Northern and Western Africa and the lowest rates are in South-Central Asia and Northern, Central, and Eastern Europe. Most (70.0% to 90.0%) primary liver cancers worldwide are hepatocellular carcinoma [3]. Cholangiocarcinomas (CCA) are rare in most parts of the world, except in Thailand where rates are high [4]. In Thailand and other parts of southeast Asia, CCA ranks as one of the most common cancers in males and females (Age-standardized rate, ASR 33.9, and 12.9 per 100,000, respectively) [5] due to the high prevalence of liver fluke infection [6]. CCAs arise primarily from the epithelial lining of the bile duct (intra- and extra-hepatic bile duct) and do not include malignancies in the gallbladder or the ampulla of Vater [5, 7]. The high ASRs of CCA in Northern and Northeastern Thailand are closely related to an endemic parasitic disease caused by *Ophisthorchis viverrini (O*. *viverrini)* infections known as opisthorchiasis. The International Agency for Research on Cancer (IARC) now classifies *O*. *viverrini* as a Group 1 carcinogenic agent [8]. The most significant risk factor for CCA is *O*. *viverrini* infection [9–12]. *O*. *viverrini* infection in humans primarily occurs because of the consumption of raw or undercooked cyprinoid fish, the intermediate host containing the *O*. *viverrini* larvae [13–14]. Since 1987, national opisthorchiasis control programs have been implemented to eradicate *O*. *viverrini* infection. There were three approaches for liver fluke control: (1) stool examination and treatment of positive cases with Praziquantel for elimination of the human host reservoir, (2) health education to promote the consumption of cooked fish to prevent infection, and (3) improvement of hygienic defecation to interrupt disease transmission.

This tripartite integrative approach has successfully limited the incidence of CCA [15]. The prevalence rate of *O*. *viverrini* infection is now considerably lower in Northern, Northeastern, and Central Thailand [16]. The sole curative treatment for CCA is surgical resection. We reported the declining trend in the incidence of CCA over the past 20 years and hypothesized this trend represented a real reduced risk for CCA [17].

We previously reported that the incidence rate of CCA has been decreasing since 2002, representing a real decline in the risk of CCA. The incidence of CCA is now projected to stabilize by 2025. Notwithstanding this decline, the survival of patients with CCA remains poor [18]. A previous study referenced data between 1989 and 2013; whereas for the current study, we expanded the date range to between 1989 and 2018. The current study thus set the 5-year periods differently. There has not yet been an update on the temporal trends, age-period-cohort model, or relative survival of CCA. The current study investigated the incidence trends of CCA, forecast future trends, and estimated relative survival.

## Material and methods

### Cancer registries and case ascertainment

CCA data were extracted from the Khon Kaen Cancer Registry (KKCR) (1989 to 2018). The KKCR, which began in 1985, is a population-based, high-quality data, cancer registry for the province of Khon Kaen in Northeast Thailand. The population in Khon Kaen province numbers about 1.7 million (available at URL: http://www.nso.go.th/) [19]. Case ascertainment in this registry has a coverage rating of 97% [20]. For cancer registration, the KKCR contains data on patients comprising all cancer sites following the IARC guidelines [21]. Patient information includes age, sex, date of birth, year of diagnosis, the basis of diagnosis, and histology. Population denominators were estimated from population censuses conducted by the National Statistical Office in 1990, 2000, and 2010 [22, 23]. Intercensus populations were estimated using a log-linear function between two consecutive censuses. The populations beyond 2010 to 2030 were estimated and reported by the Office of the National Economic and Social Development Board [23]. Age-adjusted CCA incidence was standardized to the Segi world population [24].

### Case definitions

The database was retrieved for all patients with CCA tumors living in Khon Kaen province between January 1, 1989, and December 31, 2018. Diagnoses were obtained using the International Classification of Diseases for Oncology, 3^rd^ edition (ICD-O-3). CCA is an ICD-O-3 diagnosis, and only cases with the coding C22.1, C24.0, C24.8, and C24.9 (excluding C24.1, Ampulla of Vater) were included [25].

### Statistical methods

#### Descriptive epidemiology of study patients

The characteristics of the patients were summarized using descriptive statistics. Means and standard deviations, medians and their ranges (minima and maxima) were used for continuous variables, while frequency counts and percentages were used for categorical variables.

### Trend analysis

#### Joinpoint

To identify significant changes in trends of age-adjusted CCA rates, we performed Joinpoint regression analysis using the statistical software Joinpoint version 4.0.1 [26]. Joinpoint regression identifies the annual percent change (APC) of incidence rates in each statistically significant trend interval.

#### Age-period-cohort analysis (APC model)

Age-period-cohort models were used to estimate the separate effects of age, period (calendar year), and cohort (birth cohort) on CCA incidence [27]. The APC model assumes a log-linear relationship between the incidence rate and age, period, and cohort, whereas incidence is assumed to follow a Poisson distribution with a mean equal to the product of age, period, and cohort effects. To deal with the identifiability issue of age-period-cohort models [27], we fitted the models with either cohort (AP-C) or period (AC-P) constrained to be zero on average with zero slope. The best-fitted models were determined based on the Akaike information criterion. Analyses were performed using the R package Epi [28]. We fixed the 1966 birth cohort and 1989 calendar year as a reference. The AP-C and AC-P models were fitted to CCA data separately by sex.

### Projections

We projected CCA incidence by sex until 2028, using three different approaches: the Joinpoint, Age-period-cohort, and Nordpred models [29].

#### Joinpoint model

Since recent trends are likely to be the best predictors of future cancer incidence, this projection was obtained by carrying forward the APC estimate from the last Joinpoint period to future years [30].

#### Age-period-cohort model

Age-period-cohort models (APC model) were used to study how age, calendar year (period), and birth-cohort correlate with CCA age-specific incidence risk. Age at diagnosis (A), year of diagnosis (P), and birth year (C) are linearly correlated (C = P-A), causing the well-known non-identifiability problem [31].

#### Nordpred model

We used the R package Nordpred to project CCA incidence [28]. Using the calibrated Age-period-cohort model, we computed incidence rates for each five-year age-group and the five-year interval [25]. For projections, we attenuated the drift in the Joinpoint model by 8% each year after the period of observation. For instance, detrending by 8% annually attenuates the linear trend by 1 − (1 − 0.08) k, where k = 1, 2…, N. The average attenuation rate for the first 5 years is 21.6%, which corresponds to the first five-year attenuation rate in the Nordpred model [25].

### Incidence analyses

Age Standardized Rates (ASRs) were calculated for each sex and were standardized using the Segi world standard population estimates [24]. Incidence rates are presented in cases per 100,000 by decade of diagnosis, sex, and age at diagnosis. Incidence rate ratios (IRRs) were used to compare the ASRs.

### Survival analyses

Survival analyses excluded cases if their basis of diagnosis was Death-certificate-only (DCO) or unknown; if they did not contain any follow-up information; or, if they had an unknown vital status. Survival assessments were based on relative survival (RS). RS was evaluated using mortality and life tables from the National Statistics Office in Thailand between 1990 and 2011. The analysis of RS rates was done using Stata release 10 (StataCorp LLC, College Station, TX, USA) [32]. RS results were computed using the Ederer II method [33], and survival functions were produced using the Kaplan-Meier survival method [34].

### Data processing

Data were recorded by CanReg 5 software provided by the International Association of Cancer Registries (IARC) [35]. The verification was performed with necessary correction, including logic, range, and internal consistency, which were checked using statistical software [32].

### Ethical considerations

The Human Research and Ethics Committee of Khon Kaen University reviewed and approved this project (registration: HE621484). Written informed consent was obtained from each of the patients in these studies. As for patient records used in this study, all of the data were fully anonymized before their use.

## Results

### Descriptive epidemiology and data quality

In total, there were 13,798 cases of CCA in the KKCR database between 1989 and 2018. For the period of diagnosis (every 5 years) by sex, the number of male CCA patients outnumbered females for various periods of diagnosis. The age at diagnosis trended to be high for both sexes: the mean age was 62.7 years (SD = 11.1) in males and 64.7 years in females (SD = 11.3). The most common stage of disease was ‘unknown staging’ (males 76.5%; n = 7,215 vs. females 75.8%; n = 3,314) and “Stage IV” (males 22.6%; n = 2,129 vs. females 23.4%; n = 1,024). Histological grading was commonly lacking (males 97.6%; n = 9,199 vs. females 97.9%; n = 4,282). The basis of diagnosis was endoscopic and radiologic evidence or morphological verification (males 9.5%; n = 899 vs. females 10.3%; n = 452) (i.e., based on either cytological or histological examination of tissue from the primary site, %MV) (Table 1).

**Table 1:** Characteristics of study participants at recruitment by sex.

Characteristic	Males		Females	
	Number (n = 9,426)	%	Number (n = 4,372)	%
**Diagnosis period**				
1989–1993	1,317	14.0	552	12.6
1994–1998	1,407	14.9	582	13.3
1999–2003	1,952	20.7	897	20.5
2004–2008	1,837	19.5	843	19.3
2009–2013	1,625	17.2	811	18.5
2014–2018	1,288	13.7	687	15.7
**Age at diagnosis (year)**				
15–19	1	0.1	-	-
20–24	4	0.1	3	0.1
25–29	13	0.1	6	0.1
30–34	53	0.5	17	0.3
35–39	139	1.5	47	1.1
40–44	335	3.5	107	2.5
45–49	591	6.3	238	5.4
50–54	1,018	10.8	379	8.7
55–59	1,367	14.5	560	12.8
60–64	1,668	17.7	712	16.3
65–69	1,577	16.7	766	17.5
70–74	1,274	13.5	646	14.8
75–79	799	8.5	505	11.6
80–84	429	4.5	256	5.8
85+	158	1.7	130	2.9
Mean (SD)	62.7 (11.1)		64.7 (11.3)	
Median (Min: Max)	63.0 (19: 96)		65.0 (21: 99)	
**Stage at diagnosis**				
Stage I	9	0.1	4	0.1
Stage II	19	0.2	10	0.2
Stage IIII	54	0.6	20	0.5
Stage IV	2,129	22.6	1,024	23.4
Unknown	7,215	76.5	3,314	75.8
**Histological grading**				
Well-differentiated	143	1.5	61	1.4
Moderately-differentiated	46	0.5	14	0.3
Poorly-differentiated	34	0.4	13	0.3
Undifferentiated	4	0.1	2	0.1
Unknown	9,199	97.6	4,282	97.9
**Basis of diagnosis**				
Death Certificate Only (DCO)	270	2.9	121	2.8
History and Physical examination	557	5.9	264	6.0
Endoscopy and Radiology	7,311	77.6	3,351	76.6
Surgery and Autopsy (No histolgy)	244	2.6	107	2.4
Specify Biochem/ Immuno. Test	145	1.5	77	1.8
Morphology verified	899	9.5	452	10.3

### Incidence analysis

#### Age-standardized incidence rates (ASR)

The respective overall ASR from 1989 to 2018 for all ages in males vs. females was 36.1 per 100 000 person-years (95% CI; 35.3 to 36.8) vs. 14.4 per 100,000 person-years (95% CI; 13.9 to 14.8). Additionally, the ASR varied by year of diagnosis, with the highest, respective incidence in male vs. female cases being diagnosed in 2002 (ASR = 60.2, 95%CI: 54.6 to 65.9 vs. 23.4, 95%CI: 20.2 to 26.6) (Table 2).

**Table 2:** Incidence by time period, all ages, and sex in Khon Kaen province between 1989 and 2018.

Year of diagnossis	Males				Females			
	n	CR	ASR	95% CI	n	CR	ASR	95% CI
1989	221	27.50	41.08	35.47, 46.69	89	10.98	14.76	11.62, 17.90
1990	277	34.31	51.21	45.00, 57.42	109	13.39	18.32	14.81, 21.83
1991	300	36.98	54.00	47.71, 60.29	110	13.45	18.02	14.59, 21.45
1992	236	28.93	41.44	36.01, 46.87	114	13.87	18.12	14.73, 21.51
1993	283	34.49	48.82	42.96, 54.68	130	15.72	20.32	16.77, 23.87
1994	281	34.04	46.71	41.10, 52.32	106	12.74	15.73	12.69, 18.77
1995	198	23.83	30.83	26.44, 35.22	71	8.48	10.21	7.80, 12.62
1996	244	29.17	38.49	33.51, 43.47	108	12.81	14.97	12.11, 17.83
1997	334	39.64	51.41	45.77, 57.05	145	17.08	19.54	16.33, 22.75
1998	350	41.22	52.75	47.09, 58.41	152	17.76	19.79	16.61, 22.97
1999	308	35.99	44.84	39.72, 49.96	152	17.62	19.08	16.02, 22.14
2000	339	39.28	47.57	42.40, 52.74	136	15.62	16.63	13.81, 19.45
2001	422	49.22	57.04	51.51, 62.57	198	22.79	23.12	19.87, 26.37
2002	461	54.09	60.24	54.63, 65.85	208	23.96	23.38	20.19, 26.57
2003	422	49.76	52.41	47.33, 57.49	203	23.38	21.69	18.69, 24.69
2004	367	43.47	43.81	39.26, 48.36	169	19.45	17.43	14.78, 20.08
2005	364	43.26	41.83	37.48, 46.18	188	21.60	18.81	16.11, 21.51
2006	387	46.12	42.83	38.52, 47.14	148	16.96	13.9	11.65, 16.15
2007	378	45.14	40.04	35.96, 44.12	177	20.22	15.95	13.58, 18.32
2008	341	40.76	34.34	30.66, 38.02	161	18.31	13.94	11.76, 16.12
2009	332	39.70	32.41	28.88, 35.94	161	18.22	13.04	11.00, 15.08
2010	318	38.00	29.43	26.16, 32.70	156	17.55	12.39	10.41, 14.37
2011	325	38.85	29.13	25.94, 32.32	162	18.20	12.06	10.18, 13.94
2012	357	42.73	30.98	27.73, 34.23	184	20.66	13.08	11.16, 15.00
2013	293	35.11	24.76	21.90, 27.62	148	16.58	10.07	8.42, 11.72
2014	268	32.14	21.6	18.97, 24.23	178	19.94	12.03	10.23, 13.83
2015	275	33.04	21.74	19.15, 24.33	141	15.80	9.13	7.60, 10.66
2016	268	32.24	20.44	17.95, 22.93	121	13.55	7.89	6.44, 9.34
2017	247	29.79	17.93	15.68, 20.18	132	14.80	8.16	6.73, 9.59
2018	230	27.81	16.57	14.39, 18.75	115	12.91	6.59	5.36, 7.82
1989–2018	9,426	37.6	36.1	35.34, 36.81	4,372	16.9	14.4	13.93, 14.78

n: number of cases; CR: crude rate; ASR: age-standardize rate; 95%CI: 95% Confidence interval

#### Joinpoint regression

Based on a Joinpoint regression analysis, the overall CCA incidence between 1989 and 2018 decreased significantly by: (a) −2.9% per year among males and females (annual percent change [APC] −2.9; 95% CI, −3.8 to −2.0, p-value < 0.001); (b) −3.1% per year among males (APC −3.1; 95% CI, −4.0 to −2.1, p-value < 0.001); and, (c) −2.4% per year among females (APC −2.4%; 95% CI, −3.6 to −1.2, p-value < 0.001).

The CCA incidence between 1989 and 2002 increased significantly by: (a) 1.9% per year among males and females (annual percent change [APC] 1.9; 95% CI, 0.2 to 3.7, p-value < 0.001); (b) 1.8% per year among males (APC 1.8; 95% CI, 0 to 3.5, p-value < 0.001); and, (c) 2.4% per year among females (APC 2.4%; 95% CI, 0.2 to 4.7, p-value < 0.001).

**Fig 1:**
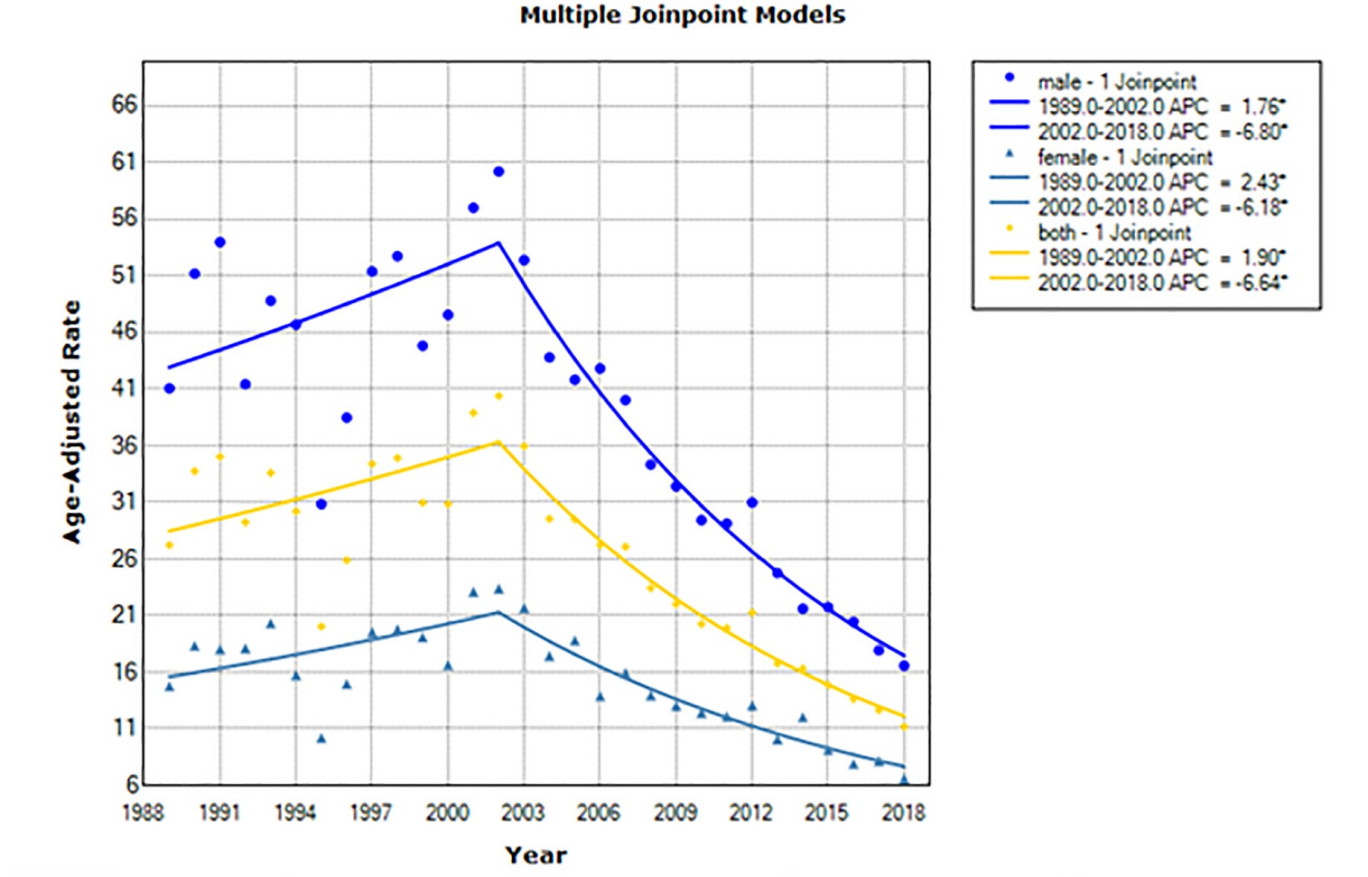
Age-adjusted incidence trends of CCA per 100,000 population in males, females, and both sexes. The lines represent the Joinpoint model predictions and the circles the observed rates in the data.

The CCA incidence between 2002 and 2018 decreased significantly by: (a) −6.6% per year among males and females (annual percent change [APC] −6.6; 95% CI, −7.8 to −5.5, p-value < 0.001); (b) −6.8% per year among males (APC −6.8; 95% CI, −7.9 to −5.7, p-value < 0.001); and, (c) −6.2% per year among females (APC −6.2%; 95% CI, −7.6 to −4.8, p-value < 0.001), (Tables 3, 4, and Fig 1).

**Table 3:** Number of cases (n) and annual percentage of change (APC) in incidence rate of CCA in Khon Kean province between 1989 and 2018.

**Characteristic**	Period	Males			Females			Both sexes		
All ages		n	APC	95%CI, p-value	n	APC	95%CI, p-value	n	APC	95%CI, p-value
	1989–2002	4,254	1.8	(0 to 3.5), <0.001	1,828	2.4	(0.2 to 4.7), <0.001	6,082	1.9	(0.2 to 3.7), <0.001
	2002–2018	5,633	-6.8	(-7.9 to -5.7), <0.001	2,752	-6.2	(-7.6 to -4.8), <0.001	8,385	-6.6	(-7.8 to -5.5), <0.001
	1989–2018*	9,426	-3.1*	(-4 to -2.1), <0.001	4,372	-2.4*	(-3.6 to -1.2), <0.001	13,798	-2.9*	(-3.8 to -2), <0.001

*AAPC: Average annual percent change

**Table 4:** Annual percentage of change (APC) in incidence rate of CCA in Khon Kaen province between 1989 and 2018.

Characteristic	Trend-1		Trend-2		AAPC
	Period of time	APC(95%CI)	Period of time	APC(95%CI)	1989–2018
**Male**	1989–2002	1.8(0, 3.5)*	2002–2018	-6.8(-7.9, -5.7)*	-3.1(-4, -2.1)*
**Female**	1989–2002	2.4(0.2, 4.7)*	2002–2018	-6.2(-7.6, -4.8)*	-2.4(-3.6, -1.2)*
**both**	1989–2002	1.9(0.2, 3.7)*	2002–2018	-6.6(-7.8, -5.5)*	-2.9(-3.8, -2)*

Abbreviations: AAPC, average annual percent change; APC, annual percent change;

*APC and AAPC are significantly different from zero; *P* < .05.

### Survival analysis

#### Overall observed survival (OS)

The five-year overall observed survival (OS) for CCA was 8.5% (95% CI: 7.9 to 8.9). Based on age at diagnosis, the respective highest and lowest five-year OS rate for age < 40 and age ≥ 61 years was 23.2% (95% CI: 18.6 to 28.1) and 7.2% (95% CI: 6.6 to 7.8). As for decade of diagnosis, the highest and lowest five-year OS rates were for 1989–1993 at 11.2% (95% CI: 9.6 to 12.8) and 2009–2013 at 4.2% (95% CI: 3.4 to 5.0), respectively. Five-year OS was higher in females than in males (Table 5).

**Table 5:** Overall observed survival (OS) of CCA for each age-group and sex in Khon Kaen province between 1989 and 2018.

*Male*											
Survival Time	Overall (%) (95% CI)	Age at diagnosis				Decade of diagnosis					
		< = 40	41–50	51–60	> = 61	1989–1993	1994–1998	1999–2003	2004–2008	2009–2013	2014–2018
1-year	17.9 (17.3, 18.7)	35.3 (29.2, 41.4)	23.0 (20.4, 25.7)	17.8 (16.3, 19.4)	16.3 (15.3, 17.3)	21.2 (18.9, 23.6)	18.0 (15.8, 20.3)	20.5 (18.6, 22.4)	16.1 (14.4, 17.8)	13.3 (11.7, 15.0)	20.0 (17.8, 22.2)
3-years	9.7 (9.1, 10.4)	25.9 (20.5, 31.7)	13.4 (11.3, 15.6)	9.0 (7.9, 10.3)	8.7 (7.9, 9.5)	11.4 (9.6, 13.4)	10.6 (8.9, 12.5)	12.8 (11.3, 14.4)	9.1 (7.8, 10.4)	4.3 (3.4, 5.3)	11.8 (10.0, 13.7)
5-years	8.2 (7.6, 8.8)	24.0 (18.7, 29.7)	12.5 (10.5, 14.7)	7.6 (6.5, 8.7)	7.0 (6.3, 7.7)	10.3 (8.6, 12.2)	9.1 (7.5, 10.8)	10.9 (9.5, 12.5)	7.1 (6.0, 8.4)	3.5 (2.7, 4.4)	10.4 (8.5, 12.5)
*Female*											
		< = 40	41–50	51–60	> = 61	1989–1993	1994–1998	1999–2003	2004–2008	2009–2013	2014–2018
1-year	20.1 (18.9, 21.4)	41.8 (30.9, 52.4)	28.4 (20.4, 25.7)	20.3 (17.8, 22.9)	18.3 (16.9, 19.8)	27.2 (23.3, 31.2)	22.3 (18.6, 26.3)	19.4 (16.7, 22.3)	18.7 (16.1, 17.8)	16.3 (13.9, 19.0)	20.9 (18.0, 24.0)
3-years	11.3 (10.3, 12.3)	26.7 (17.4, 37.0)	17.9 (11.3, 15.6)	11.8 (9.8, 14.1)	9.7 (8.6, 10.9)	16.3 (13.1, 19.8)	11.9 (8.9, 18.7)	11.3 (9.2, 13.7)	9.9 (8.0, 12.0)	8.2 (6.4, 10.2)	10.9 (8.5, 13.6)
5-years	9.0 (8.2, 10.0)	20.5 (12.1, 30.5)	16.3 (10.5, 14.7)	9.3 (7.5, 11.3)	7.6 (6.6, 8.7)	13.2 (10.3, 16.6)	13.3 (10.3, 16.8)	9.9 (7.9, 12.3)	7.0 (5.4, 8.9)	5.6 (4.1, 7.3)	10.2 (7.9, 13.0)

Overall Median survival time 20.5 (19.8, 21.3)

Overall Median survival time 23.0 (21.7, 24.3)

#### Relative survival (RS)

The five-year relative survival (RS) for CCA was 10.9% (95%CI: 10.3 to 11.6). Based on age at diagnosis, the respective, highest, and lowest five-year RS rates was under 40 years at 25.4% (95%CI: 20.4 to 30.7) and between 51 and 61 at 9.4% (95%CI: 8.4 to 10.6). As for decade of diagnosis, the respective highest and lowest five-year RS rate was 1999–2003 at 14.5% (95%CI: 13.0 to 16.2) and 2009–2013 at 5% (95%CI: 4.1 to 6.1). The five-year RS was higher among females than males (Table 6).

**Table 6:** Relative survival (RS) of CCA for each age-group, decade of diagnosis, and stratified by gender in Khon Kaen province between 1989 and 2018.

*Male*											
Survival Time	Overall (%) (95% CI)	Age at diagnosis				Decade of diagnosis					
		< = 40	41–50	51–60	> = 61	1989–1993	1994–1998	1999–2003	2004–2008	2009–2013	2014–2018
1-year	20.5 (19.7, 21.4)	38.1 (31.8, 44.4)	25.7 (22.9, 28.5)	20 (18.4, 21.7)	19 (17.9, 20.1)	24 (21.6, 26.5)	23.1 (20.7, 25.5)	24.7 (22.6, 26.8)	18.2 (16.3, 20.1)	13.9 (12.3, 15.7)	20.8 (18.5, 23.1)
3-years	11.9 (11.1, 12.6)	28.2 (22.4, 34.4)	15.1 (12.8, 17.6)	10.4 (9.2, 11.8)	11.2 (10.2, 12.2)	13.4 (11.4, 15.6)	14.3 (12.1, 16.6)	16.3 (14.5, 18.3)	10.9 (9.4, 12.5)	4.8 (3.8, 6)	13.3 (11.3, 15.5)
5-years	10.7 (10, 11.5)	26.4 (20.6, 32.6)	14.4 (12.1, 16.9)	9.0 (7.8, 10.4)	10.0 (9, 11.1)	12.7 (10.6, 14.9)	12.9 (10.7, 15.3)	14.9 (13.1, 16.9)	9.2 (7.8, 10.8)	4.2 (3.2, 5.4)	12.9 (10.6, 15.5)
*Female*											
		< = 40	41–50	51–60	> = 61	1989–1993	1994–1998	1999–2003	2004–2008	2009–2013	2014–2018
1-year	22.7 (21.4, 24)	44.6 (33.5, 55)	31.5 (26.7, 36.4)	22.2 (19.5, 24.9)	21 (19.4, 22.6)	29.7 (25.6, 33.8)	27.2 (23.3, 31.4)	24.3 (21.2, 27.4)	20.7 (17.9, 23.7)	17.1 (14.5, 19.8)	21.6 (18.5, 24.9)
3-years	13.4 (12.3, 14.6)	28.7 (18.9, 39.2)	20 (15.8, 24.5)	13.1 (11, 15.5)	12 (10.7, 13.5)	18.5 (14.9, 22.4)	19.1 (15.4, 23.2)	14.7 (12.1, 17.6)	11.5 (9.3, 14)	9.1 (7.2, 11.4)	11.9 (9.3, 14.9)
5-years	11.4 (10.3, 12.6)	22.1 (13.2, 32.5)	18.3 (14.3, 22.8)	10.5 (8.5, 12.7)	10.4 (9, 11.9)	15.9 (12.4, 19.9)	17.6 (13.8, 21.8)	13.6 (10.9, 16.5)	8.7 (6.7, 11)	6.6 (4.9, 8.7)	12.4 (9.5, 15.7)

Overall Median survival time 22.0 (0.21, 0.23)

Overall Median survival time 25.0 (24.0, 27.0)

### Trends analysis

#### Joinpoint analysis

Fig 1 shows CCA trends by sex from the Joinpoint analysis. The corresponding annual percent change (APC) estimates are presenteed in Table 3. CCA incidence significantly decresed in males from 2002 to 2018 (APC, -6.8%; 95%CI: -7.9% to -6.7%), in females from 2002 to 2018 (APC, -6.2%, 95%CI: -7.6% to -4.8%), and in both sexes from 2002 to 2018 (APC, -6.6% (95%CI: -7.8%, -5.5%) (Fig 1).

#### Age-period-cohort analysis

**Fig 2:**
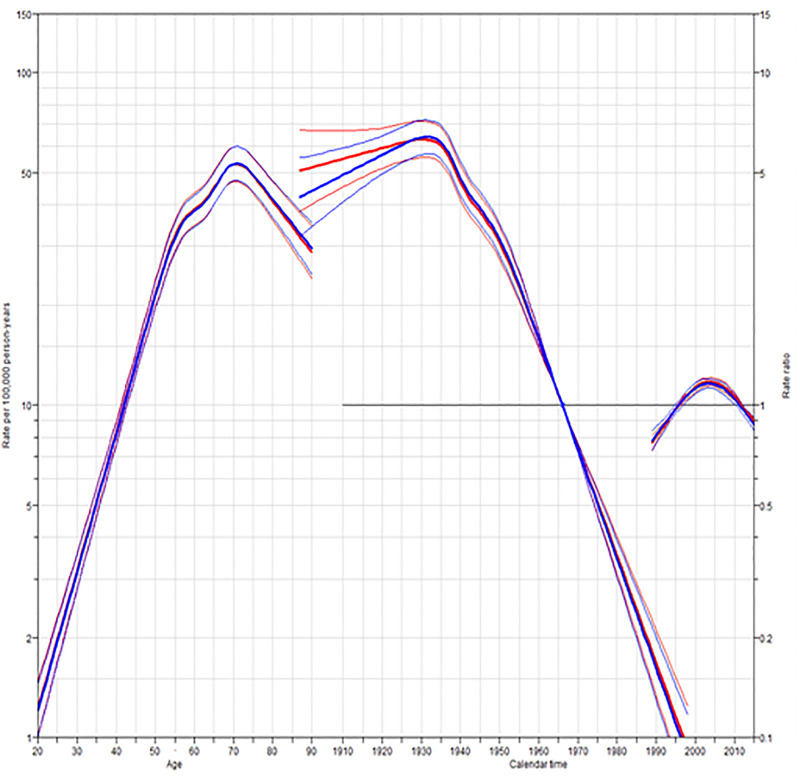
Age-period-cohort trend analysis fitted with period for CCA in males. PY, person-years.

**Fig 3:**
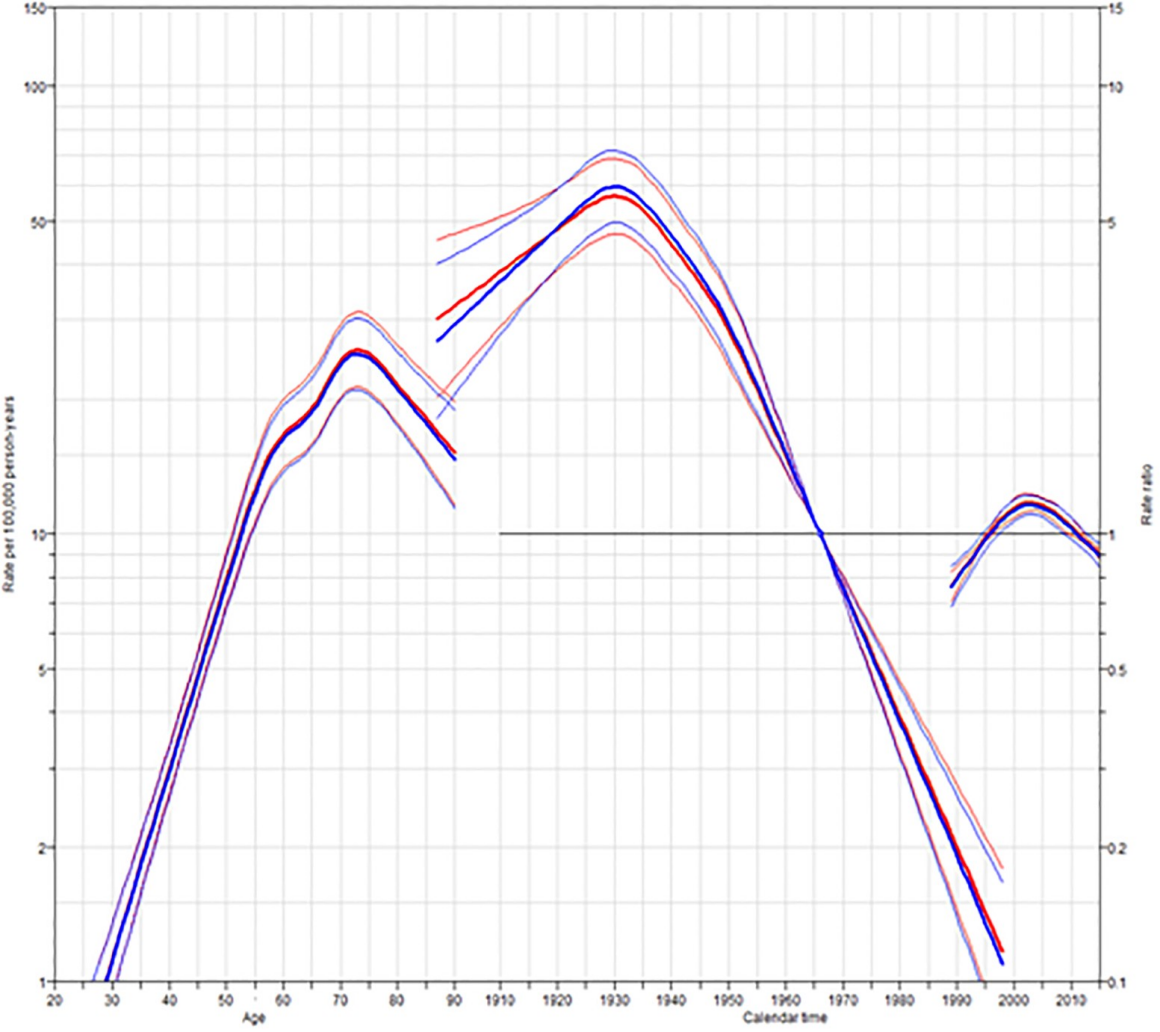
Age-period-cohort trend analysis fitted with period for CCA in females. PY, person-years.

Figs 2 and 3 show estimated age and cohort effects from the AC-P models. The CCA period effects decreased from 2000 to 2010 for both males and females, whereas the effects were relatively flat for other factors.

The age effects estimated by AC-P models of CCA trended similarly for both males and females, decreasing consistently by age. The CCA incidence rate for males increased from 1.3 per 100,000 at age 20 to 53.2 per 100,000 at age 71, while among females, it increased from 1 per 100,000 at age 20 to 25.2 per 100,000 at age 73. The AC-P model for male CCA estimates continuing increases in incidence according to a birth cohort. Specifically, the CCA incidence for males born in 1998 was 0.09 times higher than those born in 1966 (IRR = 0.09, 95%CI: 0.07 to 0.12). The relative incidence of female CCA similarly increased according to a birth cohort (IRR = 0.11,95%CI: 0.07 to 0.17). The period effects stay close to a relative risk of 1, which is consistent with the AC model that fits the data without the need for any period effects.

### Projections

**Fig 4:**
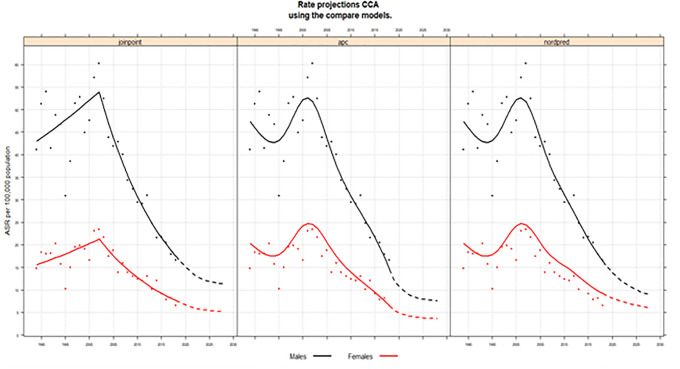
Age-adjusted incidence rates of CCA in males and female until 2028 using three projection models: Joinpoint, age-period-cohort, and Nordpred. The solid lines represent the model predictions, the dots the observed rates, and the dashed lines the model projections. AC-P, age-period-cohort trend analysis fitted with period.

Fig 4 shows the projected incidence rates of CCA by sex. Using the Joinpoint approach for CCA, the 2028 incidence rates are projected to decrease to 11.4 per 100,000 in males and 5.2 in females. As for the AC-P model project, the respective incidence rates for males and females in 2028 will reach 7.6 per 100,000 (102 patients) and 3.6 per 100,000 (140 patients). The Nordpred model projects that the respective incidence rate of CCA for males and females will continue to decrease to 8.8 per 100,000 (152 patients) and 5.9 per 100,000 (99 patients).

## Discussion

The current study (a) investigated the incidence trends of CCA, (b) forecast future trends, and (c) estimated relative survival.

### Incidence trends of CCA

#### Joinpoint, age period-cohort, and Nordpred model

Based on Joinpoint regression, the current study showed that the incidence of CCA has been significantly decreasing by −6.8% per year among males and −6.2% per year among females. This finding is consistent with the reported incidence in our previous study [17]. The respective, overall, ASR of CCA among males vs. females was 44.3 per 100,000 (95% CI: 38.9 to 49.7) vs. 17.6 (95% CI: 14.5 to 20.7). Among males vs. females, the respective incidence from 1990 to 2009 had been significantly decreasing by -0.7% per year (Annual Percent Change, APC: -0.7%, 95%CI: -2.1% to +0.8%) vs. -0.4% per year (APC: -0.4%, 95% CI: -2.1% to +1.4%) [17, 18].

All three models used in the current study showed a decrease in the incidence of CCA until 2028. The trends in incidence of CCA in Thailand are primarily influenced by infestation of the liver fluke, *O*. *viverrini* [12, 13, 36, 37]. So, the decreasing trends in CCA incidence can be explained by decreasing *O*. *viverrini* infestation in Khon Kaen province [38]. The data from the national and local levels reveal a decreasing proportion of infection, which is consistent with the ASR for CCA in Thailand [18]. Notably, the incidence of *O*. *viverrini* infection has been decreasing over time, from >60% in 1984 to <10% after 1997 [39]. Similarly, the proportion of *O*. *viverrini* infection throughout Thailand has been decreasing. More specifically, in the Northern region, the proportion has fallen from 10.3% in 1967 to 1.8% in 2019. In the Central region, it has risen from 0.3% in 1967 to 0.9% in 2019. In the Southern region, it has risen from 0.0% to 0.1%. Most notably, in the Northeastern region, the rate has fallen from 29.0% in 1967 to 4.9% in 2019 [40].

Between 2001 and 2002, the incidence of CCA in Khon Kaen declined for an annual percent change (APC) of -6.81% for males and -6.18% for females. The decline may constitute a real decline in risk as it has been 20 years since the launch of the *O*. *viverrini* infection control program in the province [17].

The decrease in the incidence of CCA might be the result of controlling the risk factors associated with *O*. *viverrini infection*. Indeed, the incidence of *O*. *viverrini* infection has decreased over time, the declining incidence, moreover, parallels a decline in *O*. *viverrini* infection rates over the last 20 years, and since *O*. *viverrini* infection is believed to be one of the risk factors for cholangiocarcinogenesis in Thailand, [18] a process that takes decades, time was also needed to evaluate the effectiveness of *O*. *viverrini* infection control (Fig 3). Numerous government policies aimed at decreasing the rate of *O*. *viverrini* infection, including: (a) liver fluke control units, established in 1967; (b) continuous health education, also established in 1967; (c) a liver fluke control program, embedded in the 5-year National Public Health Development Plan (1987–1991) [41]; and, (d) the promotion of community health through parasitic control in seven northeastern provinces, in cooperation with the Federal Republic of Germany government, run between 1989 and 1992. The liver fluke control program continues to be an element of the National Public Health Development Plan [39, 42].

Another issue that is leading to the decline in CCA would be the use of Praziquantel (PZQ) treatment. PZQ is an antihelminthic that effectively eliminates *O*. *viverrini* infection in humans. It is used worldwide, especially in Southeast Asia. Additionally, PZQ is the only chemotherapy recommended by the WHO [42].

In Thailand, national public health programs for the control of *O*. *viverrini* infection have been in place since 1987. The campaign has relied upon stool examinations and treatment of positive cases with PZQ for eliminating human host reservoirs. Health education to interrupt disease transmission promoted cooking freshwater fish in order to interrupt infection and improving sanitation and hand hygiene [43].

#### Age-period cohort model (APC model)

Age-period-cohort models (APC model) were used to study how age, calendar year (period), and birth-cohort correlate with CCA age-specific incidence risk. CCA incidence is declining dramatically in Khon Kaen province. Newer generations in Khon Kaen have a lower risk of CCA than previous generations. For example, the AC-P model suggests that men born in 1998 have a 0.09 times higher risk of CCA diagnosis than those born in 1966. Meanwhile, the incidence of female CCA shows that those born in 1998 have a 0.11 times higher risk of CCA diagnosis than those born in 1996. The problem is that the elderly continue to eat raw fish while younger generations may have regressed to more risky dietary habits, leading to an elevated risk of CCA [44]. This finding is consistent with previous studies [45]. Consequently, the widespread Northeast Thai habit of eating raw, semi-cooked, or uncooked cyprinoid freshwater fish [45, 46] continues to put people at risk *O*. *viverrini* infection [46–48]. Our data show that the mean age of CCA diagnosis was 62.7 years (SD = 11.1), 64.7 years (SD = 11.3) in males and females, respectively. The reason for this is likely the long life cycle of about ten years, latency, and persistence of *O*. *viverrine* [49, 50].

#### Survival of CCA patients

In the current study, the five-year overall OS for CCA was 8.5% (95% CI: 7.9 to 8.9), while the five-year RS remains poor at 10.9% (95%CI: 10.3 to 11.6). Our results are consistent with two previous studies in which the respective five-year survival rate was 11.2% (95%CI: 3.7 to 23.3) [51] and 10.8% (95%CI: 4.1 to 21.4), [52]. The reality is that most CCA patients continue to present at an advanced stage at diagnosis, with jaundice and positive serum carcinoembryonic antigen [53, 54]. The survival rate remains low, so the focus has been to identify other risk factors, apart from *O*. *viverrini*, such as nitrosamine and environmental factors that could be targeted to lower the incidence. Furthermore, early detection of CCA has been shown to improve survival outcomes [55]. Therfore, efforts such as ultrasound, biomarkers should be used to increase early detection.

### Screening and intervention for CCA

Cholangiocarcinoma Screening at first focused on the possible contribution of ultrasound for cholangiocarcinoma screening, concluding that the modality should be used primarily for those with the highest risk, presenting symptoms, and/or being *O*. *viverrini* positive [56]. A large number of interventions have been devised to prevent and control liver fluke infestation through action research [57]. In one community intervention an action plan was implemented, with participation of representatives of Health.

Promotion Hospitals of the Ministry of Public Health with dedicated staff, but, also schoolteachers, independent government sponsored village health volunteers, and housewives responsible for cooking and diet selection. The plan included detailed discussions of practical proposals, their introduction, assessment, and follow-on proposals at the individual village level [58]. After a health education and communication programme using local media based on local wisdom, culture, and persons, members of the experimental group had a higher level of knowledge, a better attitude, and lower levels of consumption of uncooked fish [59]. Age and health behavior to prevent CCA were factors associated with community participation and should be emphasized in future participatory action research (PAR) [60].

### Advantages and disadvantages of trends analysis, projections methods, and survival analysis

#### Joinpoint analysis

The benefit of joinpoint analysis is that it allows us to identify changes affecting different age groups in different years by comparison, a more traditional approach (viz., age- and sex-adjusted incidence rates) would only allow us to identify changes affecting the overall population. Changes in younger age groups would be undetectable, had they existed, as the impact of older age groups on the age-adjusted incidence is much higher. To further analyze incidence due to CCA in Thailand, an age, period and cohort analysis was performed using Poisson regression with natural cubic splines with six knots for age, five knots for period (= year of death) and three knots for birth cohort [61].

#### Age-period-cohort analysis

The statistical modeling of age-period-cohort (APC) data often involves the popular multiple classification model—a model containing the effects of age groups (rows), periods of observation (columns), and birth cohorts (diagonals of the age-by-period table). However, an inherent problem with this model are the adverse effects on the results of APC modeling. A potential problem with two-factor models are the interpretational limitations due to innate characteristics of typical APC data sets, which become potential sources for error [62].

#### Nordpred analysis

The global method or Average Method is typically used for short-term projections and is based on statistical regression models proposed by Bray & Møller, 2006 [63]. Limitations: The trends, obtained from statistical regression models, may not be reliable due to recent changes in coding, interventions (e.g., screening). Moreover, in his proposed method only changes of age structure and population size are taken into account to project the number of cases [64].

#### Survival analysis

Both cause-specific survival and relative survival are potentially valid epidemiological methods in population-based cancer survival studies. The choice of method is thus specific to the population and cancer type under study, and whether the proposed respective analyses are descriptive or analytic. A comprehensive understanding of the likely biases arising from each of the two methods is necessary for appropriate study design and interpretation of study findings [65].

### Strengths of the study

The present study is the first follow up examination of CCA trends by age, period, and birth cohort in Khon Kaen province, Thailand. The trends now include the most extended and latest period of diagnosis, running between 1989 and 2018. We used three alternative projection approaches, which are based on different aspects of CCA incidence, including the age-adjusted rate, APC, and period and cohort trends. The projected trends for CCA incidence include analysis by sex in future years.

### Limitations of the study

The percentage of CCA cancers with morphology verified (%MV) is low in both males (9.5%) and females (10.3%). We suggest conducting multiple imputations to further classify the unknowns as the histological data were largely missing for CCA and other cancers. Researchers should use the method from Sriplung to determine the MVs, which were also missing for CCA and other cancers [66].

## Conclusion

Our study suggests that in Khon Kaen province, the incidence rates of CCA cancer have been decreasing since 2002 and will continue to decrease over the next 26 years according to the trends in the data from the KKCR. The five-year RS rates with CCA patients are relatively low (~ 10.9%), and there was a better prognosis among female patients under 40 years of age, during the period of diagnosis 1989–1993. The three projection models used suggest that incidence rates of CCA may continue to decrease until 2028. Further research and a screening and intervention program are needed to eradicate CCA. In addition to the general benefits which would accrue from a more accurate assessment of the scale and profile of cancer in the region, cooperation among the cancer networks in the Mekong region would enable more multi-institutional research to underpin the development of comprehensive cancer control programs appropriate for the situation in Thailand.

## Supporting information

S1 Data (XLSX)
